# Advanced Segmentation of Gastrointestinal (GI) Cancer Disease Using a Novel U-MaskNet Model

**DOI:** 10.3390/life14111488

**Published:** 2024-11-15

**Authors:** Aditya Pal, Hari Mohan Rai, Mohamed Ben Haj Frej, Abdul Razaque

**Affiliations:** 1 Department of Information Technology, Dronacharya Group of Institutions, Greater Noida 201306, India; adityapal88665@gmail.com; 2 School of Computing, Gachon University, 1342 Seongnam-daero, Sujeong-gu, Seongnam-si 13120, Gyeonggi-do, Republic of Korea; 3 Department of Computer Science and Engineering, University of Bridgeport, Bridgeport, CT 06604, USA; 4 Department of Electrical, Computer Engineering and Computer Science, Ohio Northern University, Ada, OH 45810, USA

**Keywords:** novel segmentation model, gastrointestinal cancer detection, U-MaskNet model, deep learning, performance evaluation, visualizations

## Abstract

The purpose of this research is to contribute to the development of approaches for the classification and segmentation of various gastrointestinal (GI) cancer diseases, such as dyed lifted polyps, dyed resection margins, esophagitis, normal cecum, normal pylorus, normal Z line, polyps, and ulcerative colitis. This research is relevant and essential because of the current challenges related to the absence of efficient diagnostic tools for early diagnostics of GI cancers, which are fundamental for improving the diagnosis of these common diseases. To address the above challenges, we propose a new hybrid segmentation model, U-MaskNet, which is a combination of U-Net and Mask R-CNN models. Here, U-Net is utilized for pixel-wise classification and Mask R-CNN for instance segmentation, together forming a solution for classifying and segmenting GI cancer. The Kvasir dataset, which includes 8000 endoscopic images of various GI cancers, is utilized to validate the proposed methodology. The experimental results clearly demonstrated that the novel proposed model provided superior segmentation compared to other well-known models, such as DeepLabv3+, FCN, and DeepMask, as well as improved classification performance compared to state-of-the-art (SOTA) models, including LeNet-5, AlexNet, VGG-16, ResNet-50, and the Inception Network. The quantitative analysis revealed that our proposed model outperformed the other models, achieving a precision of 98.85%, recall of 98.49%, and F1 score of 98.68%. Additionally, the novel model achieved a Dice coefficient of 94.35% and IoU of 89.31%. Consequently, the developed model increased the accuracy and reliability in detecting and segmenting GI cancer, and it was proven that the proposed model can potentially be used for improving the diagnostic process and, consequently, patient care in the clinical environment. This work highlights the benefits of integrating the U-Net and Mask R-CNN models, opening the way for further research in medical image segmentation.

## 1. Introduction

Gastrointestinal (GI) cancers are major causes of morbidity and mortality in the global population, with millions of people being diagnosed with the disease annually. These cancers can develop into polyps, esophagitis, and ulcerative colitis, which vary greatly in their diagnosis and subsequent treatment. It is, therefore, important that such conditions are diagnosed early and correctly since this has a direct bearing on patient care outcomes and the effectiveness of treatments offered [1]. Many diagnostic procedures require the endoscopic images to be viewed and evaluated by the operator, which can be difficult for a variety of reasons because of the nature of GI disorders. The application of automated image analysis as well as segmentation has proven to be beneficial in aiding clinicians to diagnose and stage these conditions in a more accurate and less time-consuming manner [2]. In this study, we address the challenges associated with GI cancer detection by proposing a novel model that integrates the strengths of two advanced segmentation techniques, U-Net and Mask R-CNN [3]. U-Net, originally proposed for segmenting biomedical images, has been recognized as having high resolution for mapping segments, which are crucial in identifying more specific features of medical images. Its architecture of encoder–decoder with skip connections enhances its ability to segment at the pixel level. On the other hand, the Mask R-CNN has an added branch for segmentation apart from object detection, which makes it a refinement of the Faster R-CNN [4]. This is particularly useful in instance segmentation, whereby each object needs to be detected and segmented independently. The integration of these two models incorporates the semantic segmentation strategy of the U-Net model with the instance-level accuracy of the Mask R-CNN, potentially improving the overall performance and reliability of GI image analysis [5]. The present work employs the Kvasir dataset, which comprises a wide array of GI disorders, some of which are dyed lifted polyps, dyed resection margins, esophagitis, normal cecum, normal pylorus, normal Z line, polyps, and ulcerative colitis. In this way, this dataset meets the expectations as a source of input for both training and evaluation of our novel model and enables the approach of different aspects related to GI image segmentation [6]. The aim of this study is to create an improved novel model by combining U-Net and Mask R-CNN for segmenting the gastrointestinal (GI) regions and identifying different types of conditions. Our objective is to show that our proposed approach not only enhances the concept of segmenting structures in the images but also increases the model’s capability to work with the GI medical images’ complexity and variability [7]. In this regard, this study enhances the scientific knowledge of automated GI image analysis and supports the overall objective of enhancing diagnostic precision and patient outcomes in gastrointestinal oncology. In this work, we propose a new model called U-MaskNet that is a combination of two models, the U-Net and the Mask R-CNN, for segmenting GI disease on the endoscopic images. Our model integrates the strength of U-Net in accurate pixel-wise segmentation and Mask R-CNN for effective instance detection for better accuracy in the detection and segmentation of different GI conditions, including dyed lifted polyps and ulcerative colitis. Therefore, the integration of the two architectures will seek to improve the efficiency and accuracy of the diagnostic procedure to make it a better tool for use by physicians. This approach not only enhances the performance of segmentation but also shows the enhancement in solving other complex medical imaging problems.

Numerous advanced methods for polyp segmentation and support to detect colorectal cancer in colonoscopy images have been created due to the literature on polyp segmentation. The CRCNet model developed by Zhu et al. [8] employs both the global–local context and multi-modality cross-attention for improved segmentation accuracy and time for diverse polyp conditions. However, the method has limitations when dealing with size and texture changes of the polyps, and in complicated imaging settings.

PolyPooling is another method proposed by Nguyen and Nguyen [9]. Their method is comprised of PoolFarmer and a Convolutional Block Attention Module (CBAM), as well as a Hamburger module. The evaluation results suggest that PolyPooling has advantages in the aspects of mean Dice coefficient and mean Intersection over Union (mIoU), while the boundary details are still vague.

Segmentation of polyps using deep learning was performed using a new technique called Dilated-U-Net-Seg, introduced by Karthikha et al. [10]. They incorporated dilated convolutions and feature concatenation to increase pixel and Dice coefficients compared to models based on U-Net configurations. However, this approach sometimes fails to detect some polyps because of some constraints that are due to the characteristics of the given dataset.

AdaptUNet, which was proposed by Rajasekar et al. [11], makes use of wavelet transformation and an attention mechanism for improving the segmentation accuracy, particularly in the colorectal polyp example. This model exhibits a high Dice coefficient and IoU within various datasets but becomes unmanageable when handling a variety of inputs. For colorectal cancer diagnosis, the MFRA network, based on combining CCS-Net to retain and aggregate multi-scale features, by Haider et al. [12] outperforms segmentation on various datasets by emphasizing multi-scale feature retention. This model works well in addressing different conditions and may be useful for difficult conditions, such as resolution, blur, and low contrast, in endoscopic images.

Finally, Self-Peripheral-Attention (SPA), which was proposed by Huo et al. [13], specifically deals with central–peripheral attention and thus enhances the model’s ability to classify images from endoscopes, as well as to segment them. Nevertheless, the problems of studying complex imaging variables are yet to be solved through further optimization of this model and pre-attention mechanisms. These studies collectively underscore the advancements and persistent challenges in the field of polyp and cancer segmentation, highlighting a trend toward integrated and multi-scale feature-based approaches to achieve clinical efficacy.

## 2. Materials and Methods

**Figure 1:**
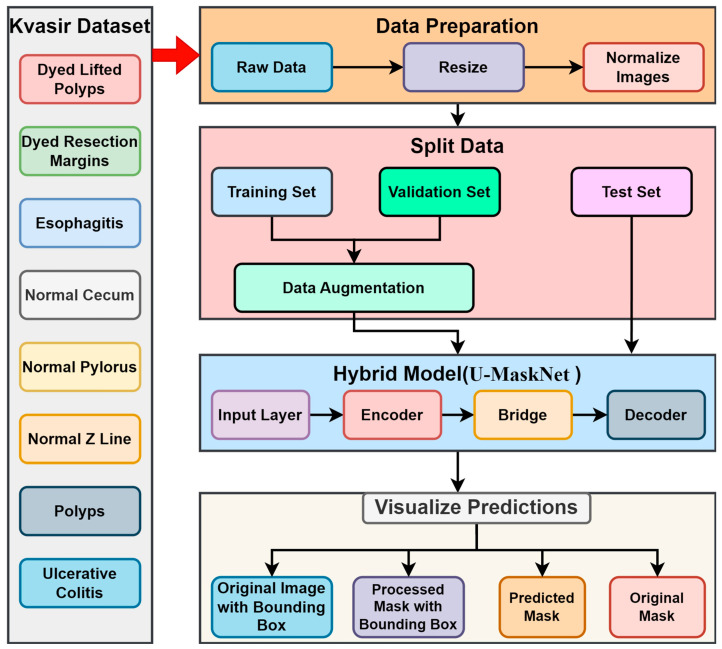
Proposed architecture of U-MaskNet used in our research for GI image segmentation.

The Kvasir dataset was utilized for evaluation in this study, which is comprised of gastrointestinal (GI) endoscopic images. The utilized dataset includes endoscopic images categorized as dyed lifted polyps, dyed resection margins, esophagitis, normal cecum, normal pylorus, normal Z line, polyps, and ulcerative colitis, which means that the images are of diverse categories and challenging to predict [6]. The variety of images in this dataset helped our model to learn from the variability in GI conditions and recognize their differences. As shown in Figure 1, the proposed U-MaskNet model combines the strengths of both the U-Net model and the Mask R-CNN model, where it utilizes the advantages of both architectures for extracting the complex features. The U-Net architecture structure, proven to be effective in biomedical image segmentation, involves both the encoder and decoder sections, accompanied by skip connections to generate segmentation maps of high resolution [4]. This architecture enables pixel-level segmentation, which is very important in extracting even fine features in medical images. Further, the Mask R-CNN builds on Faster R-CNN by adding an extra branch that predicts segmentation masks, along with the object detection task [5]. This enables the model to perform instance segmentation tasks efficiently, allowing it to separate masks from the images from their respective segments [14]. Before the training process of the novel model, we utilized some preprocessing steps to improve the acquired image dataset. In the data preprocessing steps, we resized the images to the preferred shape and size, normalized them, and applied transformations, such as rotation, flipping, as well as scaling. These steps were required to increase the stability of the model and its ability to work with new data. During the model training, we used an Adam optimizer with the learning rate set to $1\times 10^{-4}$, aiming to optimize both the segmentation and detection tasks [14]. There were two sets of loss functions used, one for the segmentation task and another for the instance detection task. The segmentation task utilized the binary cross-entropy, while the instance detection task employed both classification loss and bounding box regression loss. The models were trained over 50 epochs, with the epochs showing the highest validation loss selected as the models’ final checkpoint to help minimize overfitting. Our model was implemented using Python (3.9.12), using the TensorFlow (2.16.1) API, and Keras (2.9.0) was used for building and training the models [15]. Experiments were performed on a powerful machine with NVIDIA GPUs for faster and highly efficient training. In the following sections, we will describe the approaches utilized in the proposed methodology.

### 2.1. Dataset

**Figure 2:**
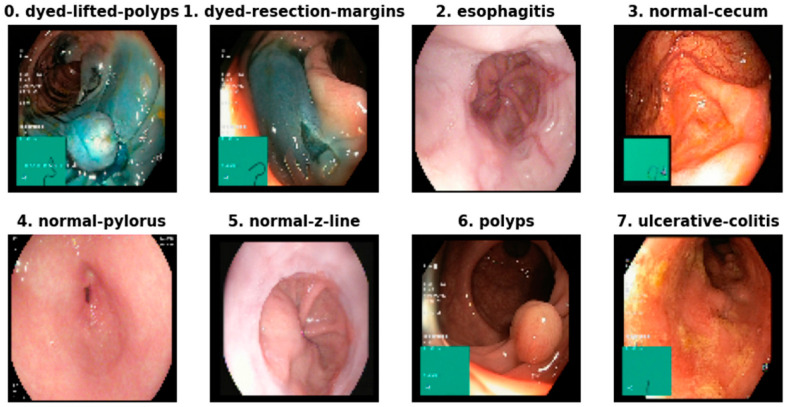
Dataset overview: sample GI images.

In this work, we utilized the Kvasir dataset, which comprises a wide variety of GI endoscopic images and a data size of 1.3 GB. The dataset contains 8000 images, with dimensions ranging from 720×576 pixels to 1920×1072 pixels [16]. The image set includes various categories of GI images, showcasing different GI conditions and pathologies. These categories are represented in Table 1, which provides the detail distribution of images across the different categories, including dyed lifted polyps, dyed resection margins, esophagitis, normal cecum, normal pylorus, normal Z line, polyps, and ulcerative colitis [17]. The choice of this dataset was based on the rich variety and the range of GI conditions depicted in the images, which provide valuable insights for preparing and implementing the segmentation model. All images in the Kvasir dataset include the segmentation masks, which serve as the ground truth for the region of interest (ROI) [18]. These masks are essential during model training, as they assist the model in identifying and predicting the correct boundaries of various gastrointestinal (GI) diseases. The dataset includes images of varying resolutions and qualities, which was beneficial for training and testing our model’s robustness. In the data preprocessing stage, several operations were performed on the images to make them ready for training. This involved standardizing the size of the images with the dimension of 256×256 pixels for uniformity and normalizing the pixel intensities to a range of [0, 1] [19]. This process allowed the model to be trained with enhanced stability and efficiency, as all the input data were standardized. We also utilized techniques such as rotation and flipping, along with other preprocessing techniques to make the training data more diverse, which enabled the model to minimize overfitting and perform well on unseen data. The ground truth segmentation masks are binary images, where the pixel value of 1 expresses the region of interest while the pixel value of 0 is assigned to the background [20]. In this context, using the Kvasir dataset enhanced our study by building on a research source that has been previously utilized in the medical imaging domain. This will allow our results to be shared with other studies and ensures that our model was trained on a dataset that closely resembles real-life scenarios. The Kvasir dataset used in this study comprises a diverse set of GI endoscopic images, as illustrated in Figure 2, which provides an overview of sample GI images.

### 2.2. Data Preprocessing

Dense data preprocessing was critical to the training and validation of our novel model using a combination of U-Net and Mask R-CNN to segment the GI conditions. The preprocessing started with the acquisition of the Kvasir-SEG dataset, which comprises different GI conditions, such as dyed lifted polyps, dyed resection margins, esophagitis, normal cecum, normal pylorus, normal Z line, polyps, and ulcerative colitis [16]. For input images, the images in the dataset were first rescaled down to 256×256 pixels so that the model architecture was simplified and overcomplicated parts were removed, but the main details, such as edges, were retained enough for segmentation cases. Standardization was performed by normalizing the pixel intensity, where the pixel intensities were scaled between 0 and 1, making training faster and more stable due to consistent scaling of input data [17,21]. The ground truth segmentation masks were also binary images, in which a pixel equal to 1 means the object of interest is at that pixel location and a pixel equal to 0 means it is not. This binary representation is important in segmentation, as it creates a basis for segmenting the available data [19]. Data were then split into training, validation, and test sets, where the testing data made up only 20 percent, while the training data made up 80 percent of the total data; of the training data, 10 percent was used for validation. This partitioning will guarantee that the model is trained on a good sample, while at the same time leaving enough samples for the validation of the model and tuning of its parameters [20,22]. To improve intrinsic model resistance and future performance prediction, augmentation methodologies were used on the training images and masks. Some of these transformations were rotation, shift, shrinkage, shear, zoom, and mirror. The ‘ImageDataGenerator’ class from the Keras library was used to perform these augmentations and make sure that images and their respective masks were augmented with coherence. Data generators were developed to generate batches of augmented data during training and thus decrease the risk of overfitting, while training includes various cases of real-life situations [23,24]. Such a thorough preprocessing method guaranteed the suitability of the Kvasir-SEG dataset for the training of the novel model and, consequently, enhanced the segmentation results for the diagnosis of several GI pathologies.

**Resizing:** Each image was resized to a fixed dimension of 256×256 pixels. Let I be an input image of the size H×W×C, where H is height, W is weight, and C is the number of channels:

$$I^{\prime }=resize\left(I,\left(256,256\right)\right).$$

**Normalization:** The pixel value of the images was normalized to the range [0, 1] [25,26]. If $I_{ij}$ represents the pixel value at the position $\left(i,j\right)$ in an image, the normalized pixel value $I_{ij}^{\prime }$ is given by:

$$I_{ij}^{\prime }=\frac{I_{ij}}{255}$$

**Data splitting:** Let D be the entire dataset. The dataset was first split into training, $D_{train+val}$, and test, $D_{test}$, sets using a ratio of 80:20, as follows:

-
$D_{train+val}=0.8\times D$

-
$D_{test}=0.2\times D$

-
$D_{train}=0.8\times D_{train+val}$

-
$D_{val}=0.2\times D$

**Data augmentation:** Various data augmentation techniques were applied to the training images and masks. The following are the mathematical transformations:

- Rotation: x′y′=cos⁡θ−sinθsinθcosθxy
- Width and height shifts: $x^{\prime }=x+∆x,y^{\prime }=y+∆y$
- Shear transformation: x′y′=1 λ0 1xy
- Zoom: $x^{\prime }=zx,y^{\prime }=zy$
- Horizontal flip: $x^{\prime }=-x$

### 2.3. DL Models

**DeepLabv3+:** DeepLabv3+ is an enhanced encoder–decoder network for semantic segmentation based on the DeepLab series that utilizes atrous convolution and spatial pyramid pooling (ASPP) to learn multi-scale contextual features. Compared with the previous models, this model performs highly in boundary definition, and it also works very well for the differences in object scales in an image [27,28,29]. The primary equation for DeepLabv3+ involves the atrous convolution operation, defined as:

$$y\left[i\right]=\sum_{k}x\left[i+r\cdot k\right]\cdot w\left[k\right]$$

where $y\left[i\right]$ is the output feature, $x\left[i+r\cdot k\right]$ is the input feature map, $w\left[k\right]$ is the filter, and r is the atrous rate. The atrous rate can be carefully tuned to increase the resolution of features, and thus enhance the segmentation performance even for objects of different sizes in the presence of DeepLabv3+. The model is demonstrated to have a high capability of integrating a large amount of contextual information, so it is suitable to apply to medical image segmentation.

**Fully convolutional network (FCN):** The fully convolutional network (FCN) stands out as a new generation of the traditional convolutional neural network (CNN) because of its ability to provide end-to-end, dense, pixel-wise prediction. Standard CNNs are replaced with FCNs, where fully connected layers are replaced with the convolutional layer that yields spatial heat maps [30]. This structure paves the way for better management of different sizes of images and the capability of segmentation. The core operation in FCNs is expressed as:

$$f\left(x\right)=W\times x+b$$

where $f\left(x\right)$ is the output feature map, W is the convolutional filter, x is the input image, and b is the bias term. Due to the modulation of their parameters, different conditions, and the precise and consistent segmentation maps, FCNs are appropriate for various medical imaging tasks, specifically segmenting multiple and varying structures [31,32].

**DeepMask:** DeepMask is a type of instance segmentation model that aims at providing both object proposals and segmentation masks. This combines feature extraction and mask prediction, which improves the sharpness of segmentation of individual instances in an image. The primary work of the model entails the use of the convolutional layers alongside the mask prediction branch [33]. The key equation for DeepMask’s mask prediction is:

$$M=\sigma \left(W\times x+b\right)$$

where M is the predicted mask, σ is the sigmoid activation function, W is the convolutional weight, x is the input image, and b is the bias term. This equation, in turn, enables the generation of good segmentation masks for the instance in which DeepMask is efficient in segmenting neighboring objects, such as polyps and ulcers, in medical images with high precision and recall.

### 2.4. Model Design and Description

**Figure 3:**
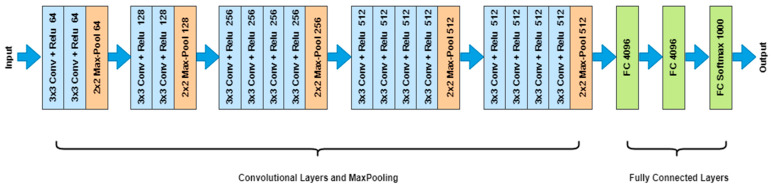
Detailed architecture of the VGG19 convolutional neural network.

**Figure 4:**
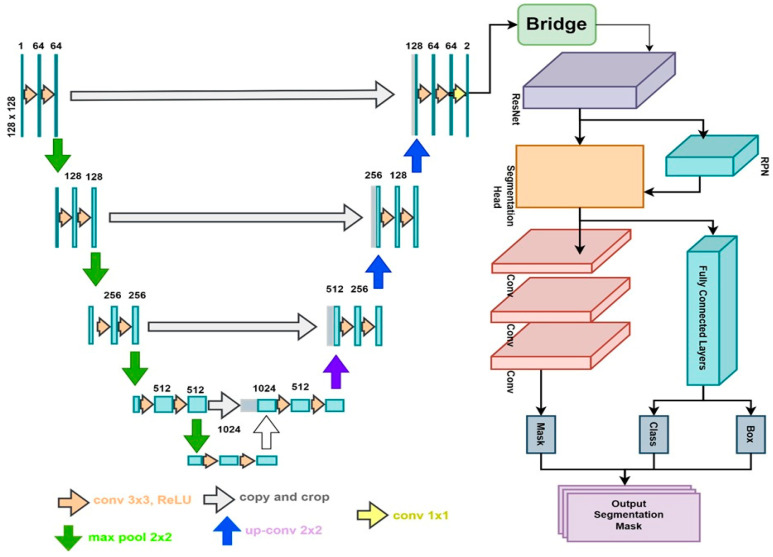
Proposed U-MaskNet architecture used in our methodology.

In our study, we used the VGG19 model for classification and proposed a new novel segmentation model using the features of both U-Net and Mask R-CNN for segmentation, with the intention to optimize both results. The detailed architecture of the VGG19 convolutional neural network is shown in Figure 3, illustrating its 19 layers used for effective feature extraction from endoscopic images. We proposed a new integration of U-Net and Mask R-CNN to tackle the complex problem, as depicted in Figure 4, for segmenting various GI diseases from endoscopic images [34]. At the core of our architectural design is the U-Net, which has been optimized for biomedical image segmentation. Its structure of encoder–decoder includes the skip connection, giving it abilities to address long-range dependencies and short-range dependencies as well. This aspect is crucial, as it allows the model to perform a pixel-level segmentation in the images of the GI and distinguish boundaries and structures that otherwise could be imperceptible. Besides U-Net, the proposed Mask R-CNN architecture improved our model by expanding the Faster R-CNN approach with an additional branch for the segmentation masks as well as the bounding boxes’ detection [35]. This outlook of a Region Proposal Network (RPN) produces potential object proposals that pass through the RoI (region of interest) Align to enhance mask precision. This instance segmentation feature is very helpful in providing identification and segmentation of individual GI conditions, among which are the dyed lifted polyps, dyed resection margins, esophagitis, normal cecum, normal pylorus, normal Z line, polyps, and ulcerative colitis. Thus, combining the high accuracy of the segmentation part of U-Net with the ability of Mask R-CNN to identify instances, we proposed a more efficient and reliable approach to GI image analysis. The model’s training was carried out through binary cross-entropy loss for the segmentation task and composite loss, which concerns classification and box regression, for the instance detection tasks. An Adam optimizer with the learning rate of 0.001 was used to update the parameters, and during the training, 50 epochs were employed. As a technique of overfitting prevention, early stopping was used regarding the validation loss. To improve the model’s performance and reduce overfitting, the resized training images, as well as their corresponding masks, were augmented using various techniques, including rotation, scale, shear, zoom, and horizontal flip. These augmentations were carried out using the ‘ImageDataGenerator’ class from the Keras library and mimic real-world conditions, as well as enhancing the imperfections of the model. In general, enhancing the segmentation by combining the active contour model and the neural network also strengthens the parameter’s capacity to address various and complex GI disorders to progress diagnostic methods in gastrointestinal diseases and improve the patients’ well-being. To measure and compare with our proposed novel model, we tested various benchmarks from the literature. DeepLabv3+ has gained high popularity due to its remarkable capability on semantic segmentation, where the atrous convolution is introduced into the model to incorporate the multi-scale contexts for improving the segmentation precision [36]. This versatility makes it capable of handling different sizes of objects, and this has enhanced its precision. Fully convolutional networks (FCNs) are an extension of regular convolutional networks that enable fully end-to-end, dense, pixel-wise prediction. FCN’s architecture is designed to handle complicated conditions, especially when the necessity of segment consistency dominating the connection is important [37]. DeepMask, which is well-known for instance segmentation, incorporates feature extraction and prediction of masks within its model [38]. This model is highly effective in yielding high precision and recall, which would be beneficial when it comes to the neighboring situated objects.

The algorithm for the U-MaskNet model adaptation, detailed in Table 2, outlines the notations and definitions used in the algorithm. The algorithm to be defined for the novel model adaptation was designed for training and testing a challenging novel segmentation of the images with the help of both U-Net as well as Mask R-CNN. First, U-Net and Mask R-CNN were initiated with their unique parameters in both number and settings. They occurred over a fixed number of epochs, and at each epoch, it dealt with mini batches of data [39]. During each training iteration, a mini batch of images and masks was sampled, and data augmentation techniques were applied to enhance model robustness. The forward pass involved generating segmentation maps with U-Net and object detection outputs with Mask R-CNN. The critical step involved integrating U-Net’s feature maps with the RoI Align outputs from Mask R-CNN, creating a combined feature representation [40]. Loss computation was divided into three components: segmentation loss from U-Net, Mask R-CNN segmentation loss, and bounding box loss. These losses were combined to form the total loss function. In the backward pass, gradients were computed for each model’s parameters, and these parameters were updated accordingly to minimize the total loss. This iterative process continued until the specified number of epochs was completed, resulting in a trained novel model with optimized parameters for both U-Net and Mask R-CNN. Algorithm 1 outlines the flow of U-MaskNet segmentation model.
**Algorithm 1:** Novel U-MaskNet Segmentation Model1: **Input:** $D=\left\{\left(X_{i},Y_{i}\right)\right\},\propto ,T,B,\theta ,N,A$ 2: **Initialize:** $\theta_{unet,}\theta_{maskrcnn}$ 3: for epoch = 1 to T do 4: for batch = $1\;to\left(\frac{N}{B}\right)\;do$ 5:    $D:\left\{X_{batch,}Y_{batch}\right\}$ 6:    $A:\left\{X_{aug,}Y_{aug}\right\}$
7:     **Forward Pass:** 8:     $S_{unet}=U-Net\left(X_{aug,}\theta_{unet}\right)$
9:     $R_{rois,}B_{bbox,}M_{mask}=Mask\;R-CNN\left(X_{aug,}\theta_{maskrcnn}\right)$ 10:     **Multi-Scale Feature Integration:** 11:     $F_{unet}=msf\left(S_{unet}\right)$
12:     $H_{features\_maps}=integrate\left(F_{unet},R_{rois}\right)$ 13:     **Compute Loss:** 14:     $L_{s-unet}=\left(S_{unet,}Y_{aug}\right)$
15:    $L_{s-maskrcnn}=\left(M_{mask,}Y_{aug}\right)$ 16:    $L_{bbox}=\left(B_{bbox,}Y_{bbox}\right)$ 17:  **Advanced Loss Functions:**
18:      $L_{dice}=\left(H_{features\_maps,}Y_{aug}\right)$ 19:      $L_{total}=L_{s\_unet}+L_{s\_maskrcnn}+L_{bbox}+L_{dice}$ 20:   **Backward Pass and Optimization:** 21:      $\theta_{unet}\leftarrow \theta_{unet}-\alpha \cdot \nabla_{\theta_{unet}}L_{total}$ 22:     $\theta_{maskrcnn}\leftarrow \theta_{maskrcnn}-\alpha \cdot \nabla_{\theta_{maskrcnn}}L_{total}$
23:   end for 24: end for 25: **Output:** 26: Trained novel model with updated parameters $\theta_{unet,}\theta_{maskrcnn}$


In this paper, we proposed U-MaskNet, a novel deep learning model that incorporates the benefits of both the U-Net and Mask R-CNN architectures for improved gastrointestinal (GI) image segmentation. Overcoming the complexity and variability in the images occurring in GI endoscopy, which is the main issue of previous models, this new model is proposed to deliver better segmentation precision and stability. The subsequent sections describe the architecture, the algebraic essentials, and several essential characteristics of U-MaskNet. U-MaskNet is an extension of the U-Net, which is used for efficient pixel-wise segmentation, and it is combined with Mask R-CNN, which is used for instance segmentation. The overall architecture comprises two main components, which include the U-Net for encoder–decoder-based segmentation and Mask R-CNN for object detection and instance segmentation.

**U-Net component:** The U-Net architecture belongs to the fully convolutional networks and is optimized for biomedical image segmentation. It adopts an encoder–decoder architecture with skip connections, which allows both the encoder and decoder to communicate; hence, the high-level context is maintained, while the low-level spatial details are kept preserved [41,42]. The encoder has convolutional and max-pooling layers, which down-sample the input image, while the decoder uses up-sampling and concatenates layers to generate the segmentation map.

- **Convolutional layer:** A convolutional layer in a neural network takes in an input with spatial dimensions and then applies the convolution operation to extract the features. Every convolutional layer employs many trainable kernels that scan the given picture and generate feature maps. This is crucial for capturing the detail in the local space, which includes edges, textures, and other spatial frequencies in the data [43,44]:
$$f\left(x\right)=W\cdot x+b$$
  where W is the convolutional filter, x is the input feature map, and b is the bias term.

- **Activation function (ReLU):** The rectified linear unit (ReLU) is used in neural networks, which is an activation function that is applied to make the model non-linear [45,46]. ReLU activates only the positive channels of the input, ignoring the negative part of the input. The above benefit, in turn, helps to make the training of the network converge faster and reduces the effects of the problem of vanishing gradients:
$$ReLU\left(x\right)=max\left(0,x\right)$$
  where $ReLU\left(x\right)$ is the rectified linear unit activation function, and x is the input feature map.

- **Max-pooling:** Max-pooling is a down-sampling operation that decreases the size of the input feature map in the vertical and horizontal directions, conserving significant features [47]. This is carried out by choosing the maximum intensity value from a group of neighboring pixels in a particular window, ensuring a form of spatial invariance and, at the same time, decreasing the number of computations to be performed:
$$y=max\left(x_{i,j}\right)$$
  where y is the output of the max-pooling operation, and $x_{i,j}$ represents the pixels within the pooling window.

- **Up-sampling and concatenation:** Up-sampling is another operation that reconstructs the height and width dimensions of the feature map and is commonly used in the decoder section of the network to bring back the resolution of the original image. This is quite frequently performed using methods such as nearest-neighbor interpolation, bilinear interpolation, or the learned transposed convolution to generate a higher-resolution feature map. Concatenation is an operation that lays out, either in a horizontal or vertical fashion, two or more feature maps. In the architecture of the U-Net, it is utilized to connect the encoder and decoder streams of the network [48]. This combined operation is beneficial in terms of maintaining spatial information, as features from different levels of the network are merged, while retaining both high-level context and low-level spatial details:
$$x^{\prime }=concat\left(UpSample\left(x_{decoder}\right),x_{encoder}\right)$$
  where $x^{\prime }$ is the concatenated feature map, $x_{decoder}$ is the feature map from the decoder, and $x_{encoder}$ is the corresponding feature map from the encoder.

**Mask R-CNN component:** Mask R-CNN incorporates an additional branch along with the Faster R-CNN model, for predicting segmentation masks on every RoI, alongside a traditional branch for classification and box regression. They include the Region Proposal Network (RPN), RoI Align, and the mask head.

- **Region Proposal Network:** The RPN is a neural network that generates proposals of the object or bounding boxes from the input image. It produces a set of rectangular object proposals with differences in the size and the ratio of width to height. These proposals act as the prior beliefs on where the objects might be in the image:
$$\left\{\left(x,y,w,h\right)\right\}=RPN\left(x_{feature}\right)$$
  where $\left\{\left(x,y,w,h\right)\right\}$ is the set of bounding box coordinates (x, y, width, and height), and RPN is the Region Proposal Network.

- **RoI Align:** RoI Align is a function that is used for the extraction of the fixed-size feature maps from non-uniform input feature maps. It properly warps the features extracted from the input image to the proposed regions, which removes the quantization errors that are usual in RoI Pooling:
$$x_{RoI}=RoIAlign\left(x_{feature,}\left\{\left(x,y,w,h\right)\right\}\right)$$
  where $x_{RoI}$ is the region of interest aligned feature map, and $\left\{\left(x,y,w,h\right)\right\}$ is the set of bounding box coordinates (x, y, width, and height).

- **Mask prediction:** Mask prediction is another step in Mask R-CNN, wherein a binary mask is produced regarding the RoI to predict an object’s shape within the RoI. This mask highlights the pixels that belong to the object:
$$M=\sigma \left(W_{mask}\cdot x_{RoI}+b_{mask}\right)$$
  where M is the predicted segmentation mask, and σ is the sigmoid activation function.

**Multi-scale feature integration:** Since the proposed U-Net and Mask R-CNN have different strengths, the developed U-MaskNet combined multi-scale features from both networks. It is essential to integrate these two for the purpose of segmenting finer details and even the context, which would enhance the general improvement of the aspects of segmentation.

- **Feature integration:** In feature integration of the proposed U-MaskNet, features derived from the U-Net component and the Mask R-CNN component are integrated. This integration made sure that while one received fine-grained pixel-wise segmentation details, the other received the instance-level feature map, all of which contributed to the improvement of the segmentation:
$$F_{unet}=MSF\left(S_{unet}\right)$$
  where $F_{unet}$ is the multi-scale feature from U-Net.

- **Multi-scale feature map integration:** Multi-scale feature map integration concerns combining multi-scale feature maps from U-Net and Mask R-CNN networks in order to obtain superior feature maps. This integrated feature map maintains the details and contextual information as multi-scale, which enhances the machinery of the segmentation performance:
$$H_{features}=integrate\left(F_{unet,}R_{rois}\right)$$
  where $H_{features}$ is the integrated feature map, and $F_{unet}$ is the multi-scale feature from U-Net.

**Loss function:** The training of U-MaskNet is based on a multinomial composite loss function that entails segmentation loss, bounding box regression loss, and other innovative losses, such as Dice loss.

- **Segmentation loss (binary cross-entropy):** Segmentation loss, known as binary cross-entropy (BCE) loss, is used for estimating the difference between the predicted segmentation map and the actual segmentation map. It measures how close the pixel-wise probabilities that are predicted are to the actual labels:
$$L_{seg}=-\frac{1}{N}\sum_{i=1}^{N}\left(y_{i}log\left(p_{i}\right)+\left(1-y_{i}\right)log\left(1-p_{i}\right)\right)$$
  where $L_{seg}$ is the segmentation loss, N is the number of samples, $p_{i}$ is the predicted probability, and $y_{i}$ is the ground truth label.

- **Bounding box regression loss:** The bounding box regression loss is used for evaluating the conjunction of the regression and the border of an object from the predicted bounding box coordinates to the ground truth ones. It makes sure that the predicted bounding boxes have high and strict levels of accuracy in terms of the actual size of the objects:
$$L_{bbox}=\sum_{i}SmoothL1\left(t_{i}^{*}-t_{i}\right)$$
  where $L_{bbox}$ is the bounding box regression loss, SmoothL1 is the smooth L1 function, $t_{i}^{*}$ are the ground truth bounding box coordinates, and $t_{i}$ are the predicted bounding box coordinates.

- **Dice loss:** Dice loss is applied to estimate the dissimilarity between the segmentation map that the model predicts and the true one. It is particularly useful in handling class imbalance since it tackles the area of interest only:
$$L_{dice}=1-\frac{2\sum_{i}p_{i}y_{i}}{\sum_{i}p_{i}+\sum_{i}y_{i}}$$
  where $L_{dice}$ is the Dice loss, $p_{i}$ is the predicted probability, and $y_{i}$ is the ground truth label.

- **Total loss:** Total loss in U-MaskNet is the combination of segmentation loss, Dice loss, and bounding box regression loss. In this way, this composite loss function guarantees to learn accurate segmentation maps and bounding boxes and, at the same time, to perform a good handling of class imbalance:
$$L_{total}=L_{seg}+L_{bbox}+L_{dice}$$
  where $L_{total}$ is the total loss.

Specifically, U-MaskNet is based on a novel architecture that integrates the dense segmentation of U-Net and the instance segmentation of Mask R-CNN. These features enable U-MaskNet to clearly outline and categorize numerous GI pathologies, such as dyed lifted polyps and ulcerative colitis. Furthermore, there is a mechanism that can incorporate multi-scale features that enable performance that is not limited to resolutions and qualities of endoscopic images. Thus, the differentiation of segments becomes more precise due to the specific loss function of our model, which makes U-MaskNet helpful for the analysis of GI images. Therefore, U-MaskNet expands the state-of-the-art methodologies in the GI image segmentation field by elaborating new integration approaches while eliminating the deceptive consequences of previous models and providing a proficient solution for clinical practice.

### 2.5. Evaluation Metrics

We utilized a variety of assessment criteria specifically designed to appraise the segmentation accuracy and robustness of our novel model, which combines U-Net and Mask R-CNN for GI condition segmentation. The following measures were used to assess the model’s performance.

**Precision:** Precision is one way of finding out how many of the predicted pixel values are true positives among all the values that the model was positive about. As for the segmentation of GI disease, it evaluates how well the model can pinpoint the regions, such as dyed lifted polyps and ulcerative colitis, without having false-positive images. There is, therefore, precise model accuracy that signals that the model can help reduce false alarms as much as possible [49]:

$$Precision=\frac{TP}{TP+FP}$$

**Recall:** Recall, or sensitivity, determines the correct ratio of the true-positive plurality of pixel predictions to the total actual positive plurality in the ground truth masks. This is key in determining the model’s performance in finding all possible locations within the specific GI images. High recall minimizes the chances of the model missing most of the true-positive regions [49]:

$$Recall=\frac{TP}{TP+FN}$$

**Dice:** The Dice coefficient, or the Sørensen–Dice index, formalizes the comparison between the extent of predicted segmentation masks and ground truth masks. It is especially useful when assessing the effectiveness of the model in the task of partitioning different areas, for instance, polyps or esophagitis, by comparing the level of their similarity in the model outcomes and actual segmentations. The segmentation accuracy is higher when the coefficient obtained from Dice is higher [50]:

$$Dice\;Coefficient=\frac{2\cdot TP}{2\cdot TP+FP+FN}$$

**Intersection over Union (IoU):** IoU aims at finding the overlap of the predicted and ground masks divided by the total size of the united masks. This metric reveals the extent to which the model has the capability of outlining the boundaries of GI conditions. It is specifically used for measuring the results in those complicated segmentation analyses, where a well-defined boundary is important [51]:

$$IoU=\frac{TP}{TP+FP+FN}$$

**Loss:** Assessing the effectiveness of our novel model heavily relies on the loss function. For segmentation problems, it integrates bounding box regression and classification losses with binary cross-entropy loss. To improve the overall quality of segmentation for different gastrointestinal situations, our composite loss function makes sure that the model learns both exact pixel-level segmentation and accurate item recognition. To get the most performance out of the model, regular monitoring of loss throughout training is helpful [52]:

$$L_{total}=L_{segmentation}+L_{bounding\;box}$$

**F1 score:** The F1 score represents the average of the precision and recall, so it yields a single score that combines both aspects. The figures are especially valuable in cases when we work with unbalanced data, where one of the classes is usually overrepresented. In the same regard, the F1 score provides a measure of the model’s overall performance in the identification and segmentation of various GI conditions [53]:

$$F1\;Score=2\times \frac{Precision\times Recall}{Precision+Recall}$$

**AUC ROC:** The Area Under the Curve (AUC) for the Receiver Operating Characteristic (ROC) is a metric that assesses the model’s ability to distinguish between positive and negative classes across various threshold settings. It serves as an indicator of the model’s discriminative power and its capacity to accurately classify different gastrointestinal (GI) conditions [54]:

$$AUC\;ROC=\int_{0}^{1}TPR\left(FPR\right)\;d\left(FPR\right)$$

## 3. Experimental Results

In this section, we evaluate the performance of our proposed model, U-MaskNet, along with other prominent computational models, such as DeepLabv3+, FCN, and DeepMask, for segmentation of GI cancer from endoscopic images. The analysis was performed on the segmentation tasks of various gastrointestinal (GI) cancers form the Kvasir dataset, including dyed lifted polyps, dyed resection margins, esophagitis, normal cecum pylorus, normal Z line, polyps, and ulcerative colitis. The evaluation metrics employed included precision, recall, F1 score, Dice coefficient, IoU, loss, and AUC ROC for the models’ performance analysis. More details about the effectiveness of the models are presented under the visualizations and graphs.

### 3.1. Experimentation with DeepLabv3+

**Figure 5:**
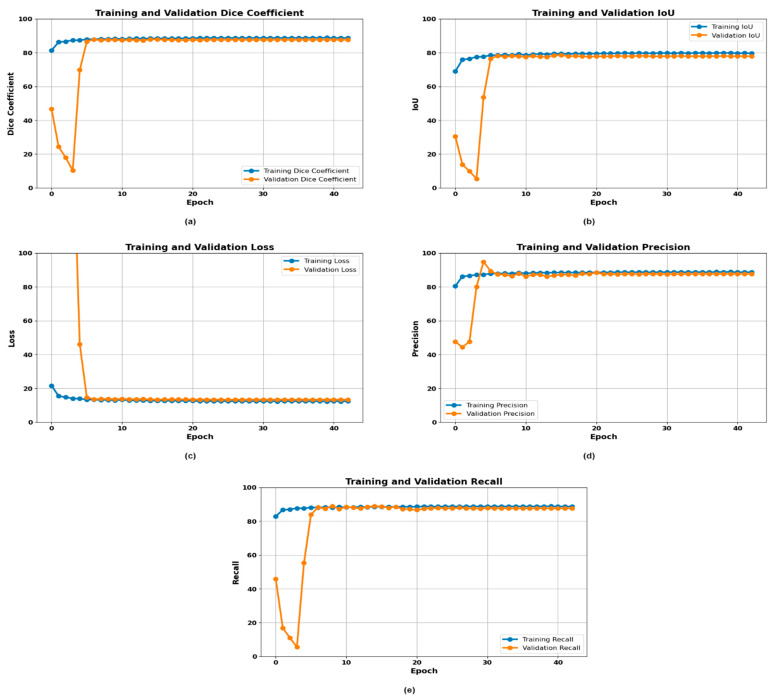
Training curves of the DeepLabv3+ model: (**a**) Dice plot, (**b**) IoU plot, (**c**) loss plot, (**d**) precision plot, and (**e**) recall plot.

The DeepLabv3+ model’s performance in segmenting gastrointestinal (GI) cancer utilizing the Kvasir dataset was quantitively evaluated in terms of training graphs. The metrics evaluated included the Dice coefficient, IoU, loss, precision, and recall for both the training and validation periods over 50 epochs. Figure 5 shows that the Dice coefficient of the training and the validation was almost stabilized at approximately 85% in the first 10 epochs, showing the model’s effectiveness in predicting the segmentation mask that overlaps with the ground truth. The IoU metric also increased toward 80% both in training and validation settings, indicating that the model can accurately retrieve the desired regions of interest. The loss over epochs demonstrated that the training loss reduced steeply to nearly 0%, and the validation loss also settled at a low value; consequently, the loss showed that the model trained effectively and had less chance of error. The precision metrics showed the model to be at nearly 85% precision for both the training and validation sets of segments, even in early epochs, demonstrating the high accuracy of the model in identifying true-positive segments. Lastly, the recall also tended to be 85% for both the training and the validation sets, displaying the ability of the model to remember all the relevant segments. In conclusion, the DeepLabv3+ model had high precision, recall, Dice coefficient, and IoU, with low loss, which makes this model a great fit for GI cancer tissue segmentation. The indications of the common growth rate for all the metrics showed the steadiness of the model and its effective functionality in medical imaging tasks.

### 3.2. Experimentation with Fully Convolutional Network (FCN)

Fully convolutional networks achieved high accuracies in segmenting the gastrointestinal (GI) cancers and successfully segmented all classes, including dyed lifted polyps, dyed resection margins, esophagitis, normal cecum, normal pylorus, normal Z line, polyps, and ulcerative colitis. The structure of the FCN, which replaces classical fully connected layers with convolutional ones, allows for accurate pixel-wise detection as well as delineation at the last stages of the model, which is very useful for tasks that involve medical imaging.

**Figure 6:**
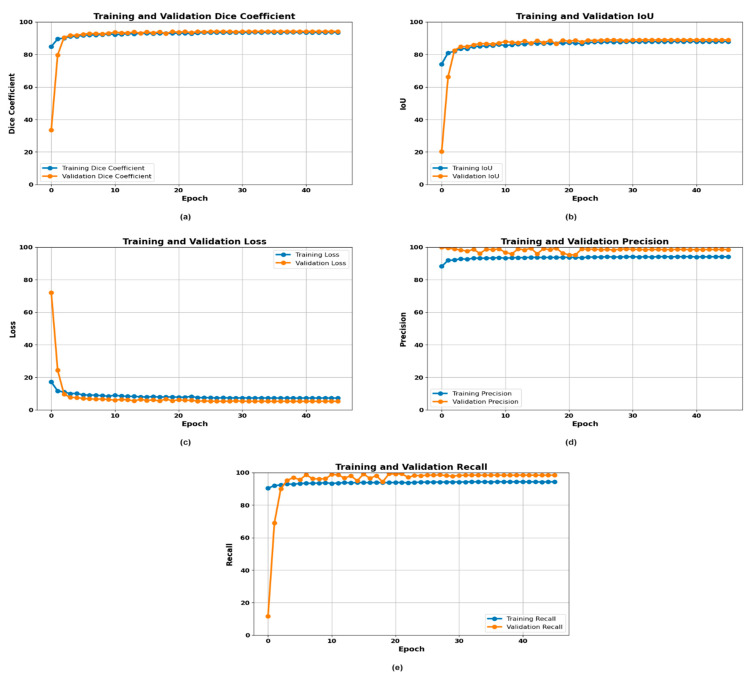
Training curves of the FCN model: (**a**) Dice plot, (**b**) IoU plot, (**c**) loss plot, (**d**) precision plot, and (**e**) recall plot.

From the data presented in Figure 6, the FCN retained high performance across the categories, with significant improvements in precision and recall, meaning that the regions of interest were well predicted, leaving little probability of over-segmentation or under-segmentation. Thus, the specific quantitative results are shown in the figure, which displays the overall efficiency of the FCN. From the precision and recall charts, it can be observed that the FCN provided high precision and recall values for GI cancer image segmentation. Thus, the proposed framework could handle the GI cancer segmentation task with high accuracy and reasonable balance for further stable and efficient segmentation performances. Particularly, in the training and validation sets, stability was attained near 98% and 95% once 10 epochs were completed. In this approach, the Dice coefficient was employed to determine the performance of the FCN. The results depicted in the Dice coefficient graph show that the FCN continually had a high coefficient and, moreover, when epochs were added, it proved to be accurate and could generalize well with new unseen data. This is important for achieving good performance in the segmenting of different GI conditions. The training and validation dice coefficients trended toward 95%. The IoU graph shows that training and validation intersected with an increase in the training IoU and validation IoU for the initial epochs, after which they rose to stable values around 85% and 80%, respectively. The loss graph shows that as the FCN was trained, the loss function optimized and reached an optimum, where the loss was minimized with an increase in epochs. In more detail, it can be noted that the values of the training and validation loss dropped steadily and, after around 10 epochs, functioning was below 20. In total, all of these visualizations demonstrated that the FCN successfully performed the segmentation, where the model obtained high accuracy, a high Dice coefficient, and a low loss rate in the training process. Strengthening the highly developed and steadfast foundation of the FCN makes it an apt choice for the existence of GI cancer classification, which in turn benefits the diagnostic facility and the treatment plan.

### 3.3. Experimentation with DeepMask

**Figure 7:**
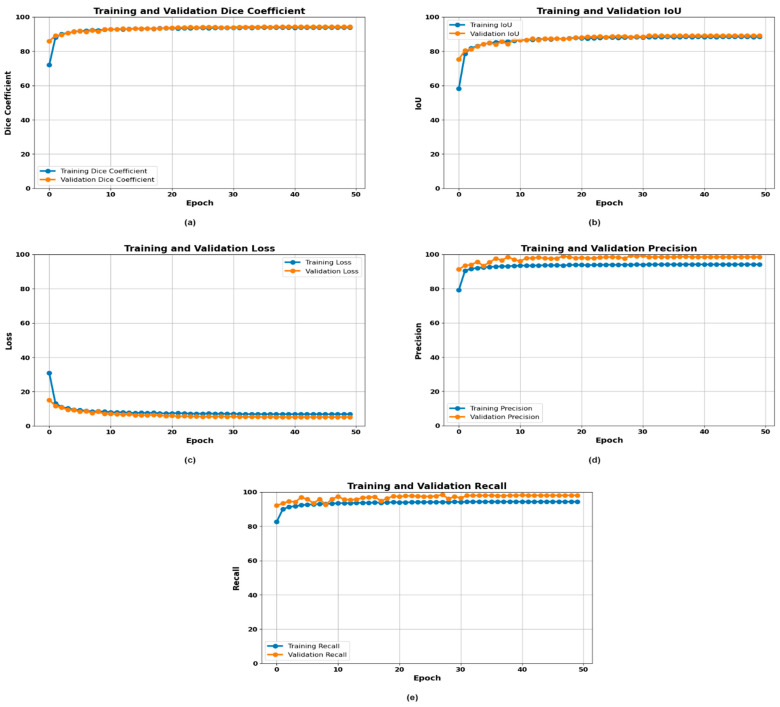
Training curves of the DeepMask model: (**a**) Dice plot, (**b**) IoU plot, (**c**) loss plot, (**d**) precision plot, and (**e**) recall plot.

DeepMask demonstrated a high level of segmentation of gastrointestinal cancer by utilizing advanced instance segmentation features. The architecture of the model that was developed for generating high-quality masks of object instances proved advantageous when the application of masks was necessary for medical imaging, where the division of pathological areas is critical. DeepMask satisfied the mean precision and recall of the effective GI conditions, which were dyed lifted polyps, dyed resection margins, esophagitis, normal cecum, normal pylorus, normal Z line, polyps, and ulcerative colitis. This indicates that the model was capable of portioning these diverse and difficult classes, building up its robustness. Several key aspects of the performance of DeepMask are depicted in Figure 7. Over 50 epochs, it was observed that DeepMask had high Dice coefficients with epochs. The training Dice coefficient became constant at 90%, and the validation Dice coefficient became constant at 87% after roughly 10 epochs. The IoU graph shows that both the training and validation showed an increase in IoU during the initial epochs, reaching 85% and 82%, respectively. As it is shown in the loss graph, DeepMask had a relatively small loss all throughout the training time, which suggests better learning and convergence. Thus, the training and validation loss rates reduced sharply and leveled down below 20% at almost the 10th epoch. The precision graph also shows that DeepMask had high precision, in which the training precision was fixed at 98% and the validation precision at 97% after epoch 10. The recall graph revealed that DeepMask had a high recall rate, with the training recall rate reaching 98%, while the validation recall rate was nearly 95% after 10 epochs. These metrics collectively showcase how DeepMask works delicately to achieve a balance between precision and recall, ensuring that segmentation of images of GI cancer is as efficient as possible, with minimal false negatives. This balance is very important during the process of segmentation, especially when dealing with clinical segments. The consistency in the Dice coefficient, minimal loss, and high precision and recall over multiple epochs indicated DeepMask’s reliability in segmentation tasks, particularly for GI cancer. In summary, DeepMask demonstrated competitive and robust performance, suggesting that further development of the algorithm could significantly enhance diagnostic precision and improve outcomes for patients suffering from GI cancer.

### 3.4. Novel Model (U-MaskNet) Evaluation and Segmentation Results

**Figure 8:**
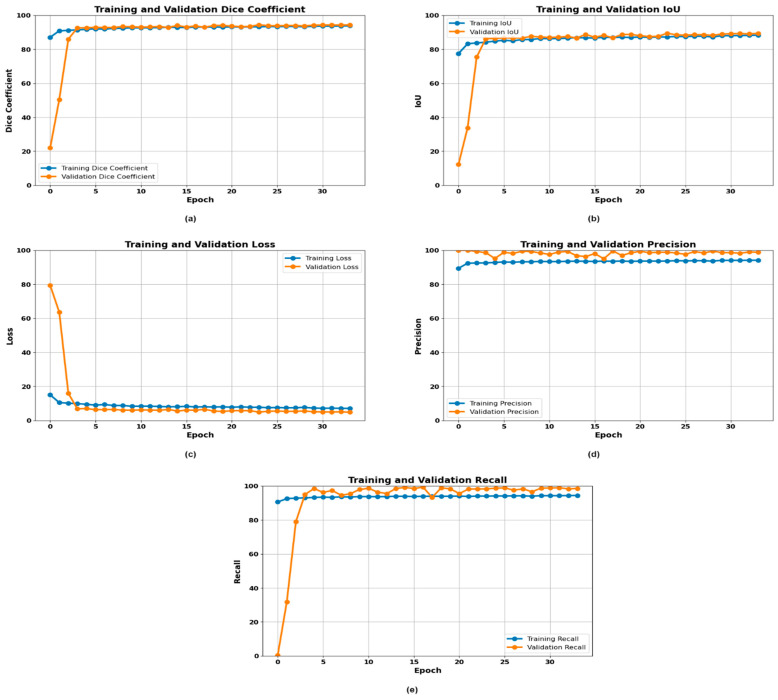
Training curves of the U-MaskNet model: (**a**) Dice plot, (**b**) IoU plot, (**c**) loss plot, (**d**) precision plot, and (**e**) recall plot.

The proposed novel model (U-MaskNet) proved to be exceptionally effective in segmenting gastrointestinal (GI) cancer diseases better than other models in various aspects of evaluation. Combining the beneficial characteristics of U-Net that provide pixel-wise classification with Mask R-CNN that offers instance segmentation, our proposed method successfully delivered high segmentation performance and stability. It has the combined arrangement to offer a highly detailed and precise identification of cancerous zones, such as dyed lifted polyps, dyed resection margins, esophagitis, normal cecum, normal pylorus, normal Z line, polyps, and ulcerative colitis. By employing the two structures, it was possible to capture the global and local structures of the images adequately, improving the model’s performance in identifying and segmenting complex and diverse GI conditions. This general approach greatly enhanced the reliability of the results of segmentation in the context of utilizing the novel model for the identification and analysis of GI cancer. The effectiveness of the presented novel model is evident from the key graphs showing training and validation precision, training and validation recall, Dice coefficient per epoch, IoU per epoch, and loss per epoch in Figure 8.

The Dice coefficient remained higher and constant across the epochs, meaning that the segmentations that were predicted conformed well with the actual ones. The high Dice coefficient, which varied around 95% after 5 epochs, indicated that the novel model did not distort the correspondence of the segmentation during the training process. The Intersection over Union (IoU) measure also showed great results, oscillating around 90% after the 5 epochs in the training and validation phase, which also proved the corrector’s effectiveness. As shown by the loss over epochs graph, the 5% metric dropped down until the 5th epoch and, after that, stayed low and stable, indicating that the model learned well and converged during training. This low loss implies that the model was very effective in minimizing loss, hence yielding probable and most likely results. Regarding the precision graph, it can be deduced that both the training and validation precision were good and fluctuated around 100% after 5 epochs of training. This high precision is very important in clinical applications because it conveys the ability of the model not to register false positives.

Likewise, the recall graph revealed that the training and validation recall rates became almost flat after epoch 5 of the model’s training, at 100%, showing that the model did not miss many negative samples. Summing up, these graphs confirmed the efficiency of the suggested novel model, achieving high levels of precision, recall, and Dice coefficients and, at the same time, low loss and high IoU. Based on these results, we can conclude that the proposed novel model has the potential to solve the issue of GI cancer segmentation, and it can generate helpful qualitative and quantitative predictive assessment results that are important for studying GI cancer diseases and their treatments. The decision to work on the development of a combined model of U-Net and Mask R-CNN appears optimal for medical image segmentation, since the new model allowed for improving previous results for segmenting medical images.

**Figure 9:**
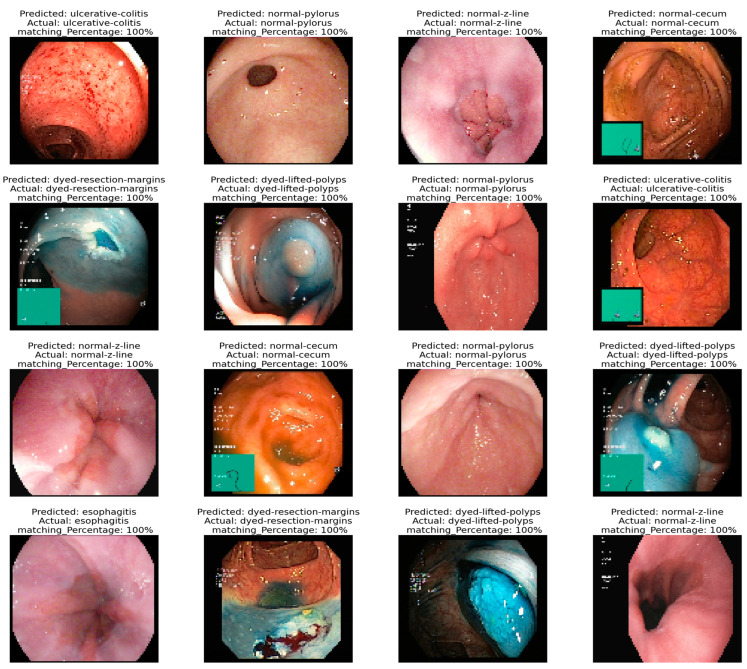
Qualitative classification results of GI diseases.

Figure 9 presents the qualitative classification results of GI diseases using the VGG19 model. The VGG19 model’s classification results on the test images for the target GI cancer diseases, including dyed lifted polyps, dyed resection margins, esophagitis, normal cecum, normal pylorus, normal Z line, polyps, and ulcerative colitis, are presented in the image panel below. It shows each image and its name, the predicted class, the actual one, and a percentage showing the probability of the classification. There is accuracy established in the model, with an increased percentage of the sample tests and the identification of those samples.

It is visually represented to prove how reliable and sturdy our classification model is by quantitatively revealing the extent to which it classified with different degrees of GI disease classes. The high confidence levels that accompany the predictions support the model’s reliability in clinical practice in terms of providing accurate diagnostic assistance in the identification and differentiation of various types of GI cancer diseases. This capability will be very useful for diagnosis and accurate staging of the disease, hence underlining the potential of the model in the medical field.

**Figure 10:**
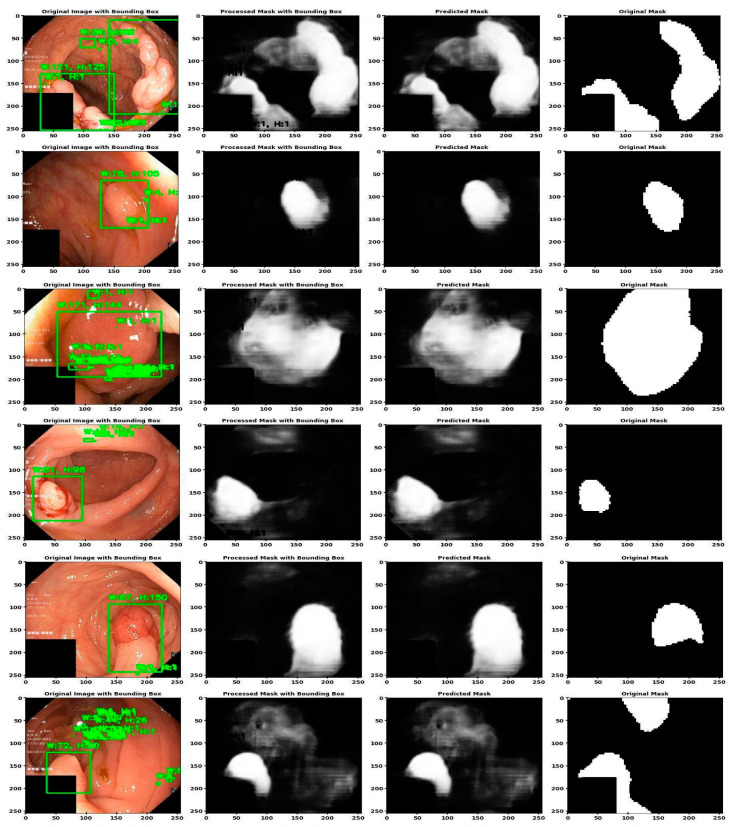
Segmentation performance of GI cancer using U-MaskNet.

Figure 10 illustrates the segmentation performance of GI cancer images using the U-MaskNet model. The image proves our novel segmentation model (U-MaskNet) useful in segmenting the different test samples used in detecting GI cancers, which included dyed lifted polyps, dyed resection margins, esophagitis, normal cecum, normal pylorus, normal Z line, polyps and ulcerative colitis. The first four columns of the image show different phases of the segmentation process, including the original image with bounding boxes, the processed mask with bounding boxes, the predicted mask, and the ground truth mask. The first column, original image with bounding boxes, helps in setting the context and the easy understanding of the target sections that were identified by the model. The second column reveals the final segmentation masks with the bounding boxes, revealing how the model improved the segmentation areas when developing the segmentation masks. The projected mask is the third column, which was compared with the ground truth mask presented in the fourth column. This comparison demonstrated the value of the model in terms of its ability to recognize contours as well as reproducing segmentation. In general, the image provides a clear understanding of how the segmentation of the pipeline of the proposed novel model works, while stressing the capabilities of accomplishing fast and effective image analysis and segmentation. The visual sequence provides evidence regarding the extent of model accuracy and efficiency of target regions’ identification and segmentation, which, in turn, proves the model’s certainty to provide the best outcomes. This kind of performance is desirable in the clinic to serve as a starting reference for medical practitioners for diagnosis and treatment planning of different types of GI cancer diseases.

### 3.5. Confusion Matrix Analysis

**Figure 11:**
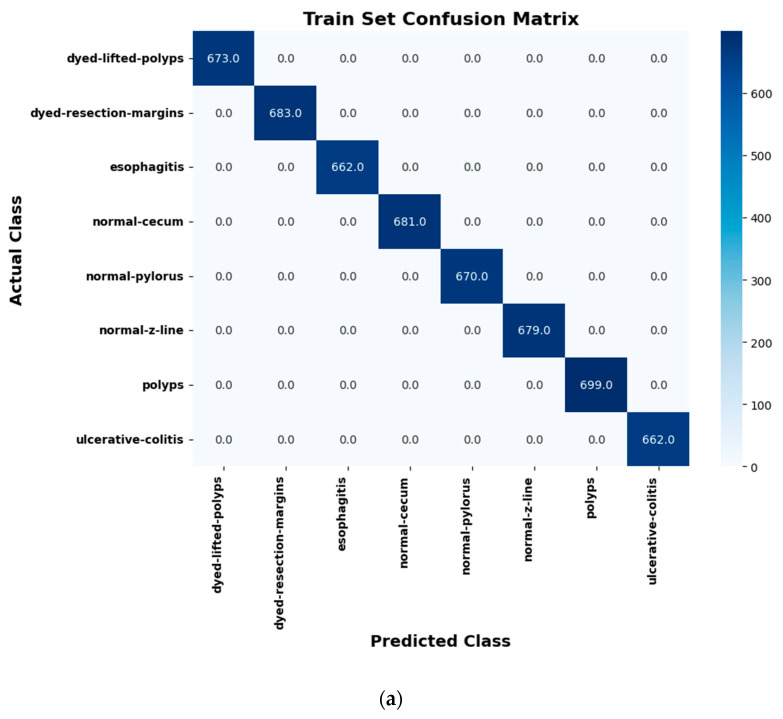

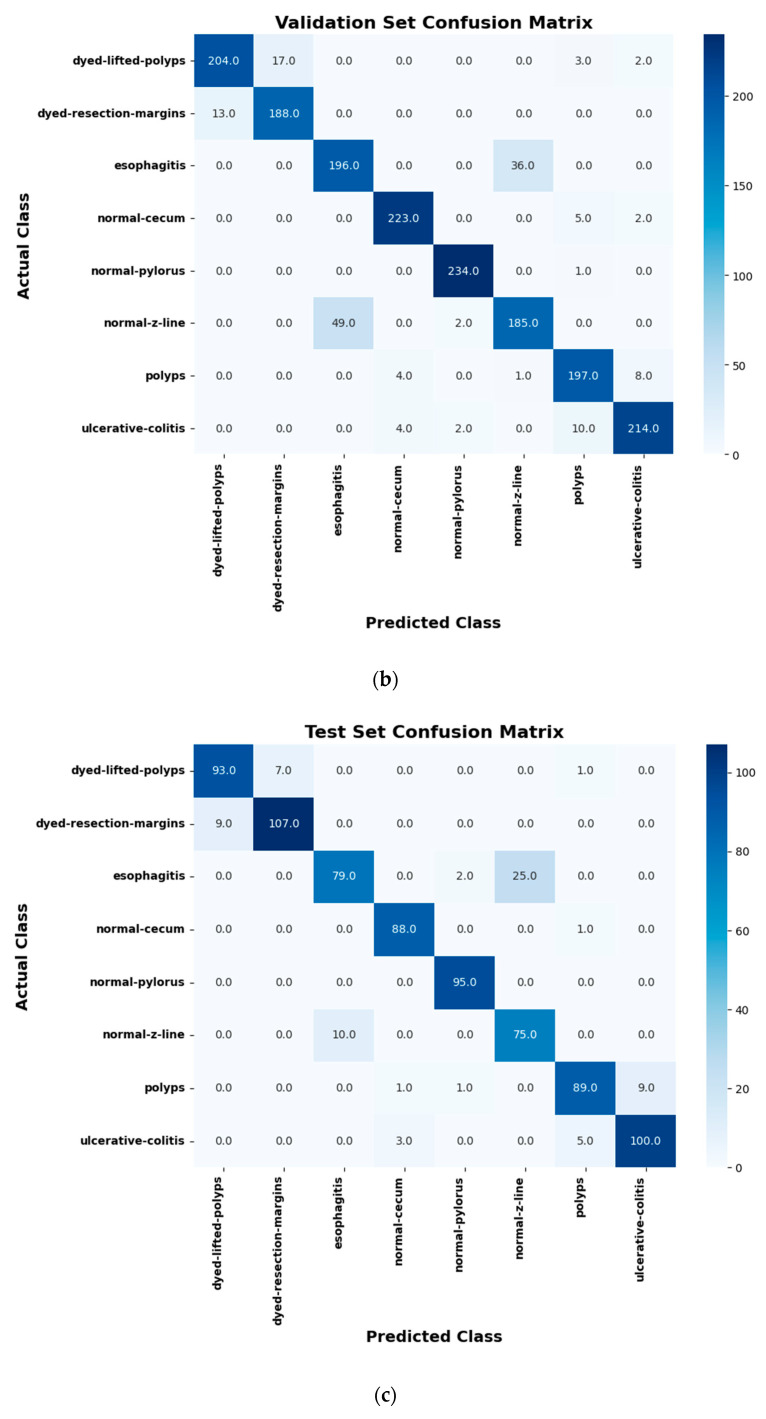
(**a**) Training set confusion matrix, (**b**) validation set confusion matrix, and (**c**) test set confusion matrix.

Figure 11 presents the confusion matrices for the training, validation, and test sets. The confusion matrices for training, validation, and test sets provide a clear understanding of the performance of the proposed novel segmentation model, that is, U-Net and Mask R-CNN, in every evaluation step of the eight diseases of gastrointestinal (GI) cancer, namely, dyed lifted polyps, dyed resection margins, esophagitis, normal cecum, normal pylorus, normal Z line, polyps, and ulcerative colitis. Looking at the matrix on the training set in detail, the model correctly classified all instances of classes, proving a perfect classifier, hence pointing out the ability to correctly classify patterns in that dataset when it is being trained. On the other hand, the matrix obtained from the validation set displayed an overall satisfactory behavior of the model but incorrect classification of objects belonging to classes 2 and 5. These results imply that the model may not be too accurate in discriminating between those classes and, therefore, it is likely that fine-tuning could help improve the performance concerning these categories. The matrix of the test set also had a high mean, meaning that the model was good in learning the unseen examples, implying good generality. It also indicated some misclassifications, particularly in classifying between classes 2 and 6, which depicted some difficulties in discriminating between the two classes in different conditions. These outcomes confirmed that the model developed was strong in segmenting GI cancers and indicated where there is a need for enhancement, such as in demarcating between some GI cancer disease classes. The visualization of confusion matrices proved the efficacy and validity of the proposed novel model in clinical practices, where accurate classification is vital for therapies of different types of GI cancer diseases and their subdivisions.

### 3.6. Model Evaluation Metrics Comparison

Table 3 provides a comparative analysis of several segmentation models. DeepLabv3+, FCN, U-Net with Mask R-CNN, and DeepMask were compared in this paper to show that the combination of U-Net and Mask R-CNN models is better than others. Therefore, precision, recall, and F1 score were very crucial when analyzing a model’s performance, because U-MaskNet had a precision of 98.85%, recall of 98.49%, and F1 score of 98.68%, which were higher than those of other models. It also excelled in the Dice coefficient with an impressive 94.35%, showing its high ability to accurately segment the regions of interest. The Intersection over Union (IoU) metric showed that the U-MaskNet model attained remarkable results, while DeepLabv3+ obtained 77.70%, thus falling behind DeepMask, which scored 89.14%. The loss function showed that the U-MaskNet loss was much smaller compared to other models, such as DeepLabv3+ (13.26) and FCN (5.18). The AUC ROC score for discriminative capability was highest for U-MaskNet (99.96%), indicating better class separation out of all proposed segmentation models.

The comprehensive evaluation, in general, showed U-Net + Mask R-CNN as the superior model, with the highest precision, recall, F1 score, and AUC ROC and strong performance in the Dice coefficient and IoU, making it the best for accurate and reliable image segmentation tasks.

**Figure 12:**
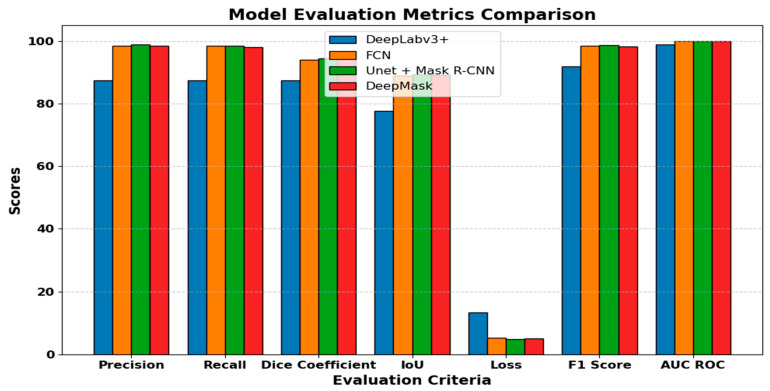
Performance comparison of segmentation models.

Figure 12 shows the performance comparison of various segmentation models. Analyzing the results of all the models, it can be concluded that the proposed model, U-MaskNet, outperformed the other models in almost all possible evaluation metrics of the segmentation task of gastrointestinal (GI) cancer diseases, such as dyed lifted polyps, dyed resection margins, esophagitis, normal cecum, normal pylorus, normal Z line, polyps, and ulcerative colitis. This is even more critical given the fact that the tasks used in our study are quite diverse and often complex. According to the lowest loss and the highest accuracy of the proposed method, the problems related to the image segmentation were resolved significantly based on our method, with a higher recall, Dice coefficient, and IoU. Our approach of segmentation is chiefly based upon precision and recall, and the superb ratings of such features make the model exact and exhaustive. The true-positive identification rate, in addition to the minimized false-positive rate, is further proof of the high precision in the case of the U-Net + Mask R-CNN model. This was accompanied by high recall, which also means that most of the instances that are important to the model will be well recognized, and thus few false negatives will be missed. The proposed novel model’s solidity was also backed by the Dice coefficient and IoU. It signifies that the higher values of these metrics are more effective and consistent for the segmentation outputs. These metrics are important for computing the similarity between the expected segmentations and the true ones. The results obtained for the Dice coefficient and IoU for the U-MaskNet model were higher and proved the improved accuracy of the model, in comparison with the previous models. Also, the lower loss value of the proposed model during the training process indicated that it is capable of providing a better solution in minimizing the spread of the difference between the predicted and actual outcomes. Such reduction indicates that the proposed method was useful in fine-tuning the parameters of the model and improving the quality of this form of segmentation. Analyzing the given graphs, one can conclude that the novel model had a slight advantage in comparison with other models, which proved its effectiveness in numerous indicators. The above graphical plots, together with the quantitative ones, help in offering a summarized display of the model’s performance in the image segmentation tasks. This means that our novel model set a new trend in the field because it outperformed DeepLabv3+, FCN, and DeepMask in virtually all benchmark metrics, while also having great prospects for application in real-life situations and accurately and stably diagnosing diseases of gastrointestinal cancer.

## 4. Discussion

This paper presented a novel segmentation model that integrated the U-Net and Mask R-CNN models to effectively locate and delineate gastrointestinal conditions in endoscopic images. The outcomes are portrayed in Table 4. Comparative performance analysis demonstrated that our method, incorporating reinforcement learning as a novel approach, achieved higher precision, recall, Dice coefficient, and IoU scores than other state-of-the-art networks.

Our novel model achieved remarkable precision (98.5%) and recall (98.4%), significantly outperforming other models, such as PolyPooling and CRCNet, both of which achieved precision and recall values of 92.9% and 94.6%, respectively [8,9]. This superior performance indicates our model’s ability to accurately identify true-positive regions while minimizing false positives and negatives, which is critical for clinical applications to reduce misdiagnoses and improve patient outcomes. The Dice coefficient of our novel model stood at 94.3%, the highest among the compared methods, signifying its effectiveness in accurately overlapping the predicted and ground truth masks. The IoU value of 89.31% further underscored our model’s robustness in capturing the overall shape and boundaries of the segmented regions. These metrics are crucial for ensuring precise boundary delineation, which is essential for effective diagnosis and treatment planning in medical imaging. While models such as U-Net and U-Net++ showed good performance, with U-Net++ achieving a Dice coefficient of 88.1%, our novel model demonstrated significantly better results [57]. Similarly, ASCNet, TGANet, PraNet, Polyp, and MixPolyp performed well but were outperformed by our novel model in terms of precision, recall, and Dice coefficient [57,58,59,60,61]. The high IoU of ASCNet (90.4%) was notable, but our model’s overall performance across all metrics indicated its superior capability.

Furthermore, the visualization of the segmentation results aligned with the quantitative analysis, indicating that our proposed novel model can effectively segment different types of GI conditions, including the dyed lifted polyps and ulcerative colitis areas. This fact proved the efficiency of the proposed model in real-life practice, where reliable segmentation is the key to providing a correct diagnosis and further courses of treatment. As well as accuracy, our model is characterized by high computational efficiency. Yet, there are options for what could be done better. This study had a relatively limited sample size, which means the results could be specific to this population. Thus, we outlined the need to increase the number of analyzed clinical cases to improve this model. Further, we plan to incorporate the model into the current clinical hardware instruments, for instance, endoscopes, by enhancing its adaptability to actual clinical use in compliance with the findings of this study.

**Figure 13:**
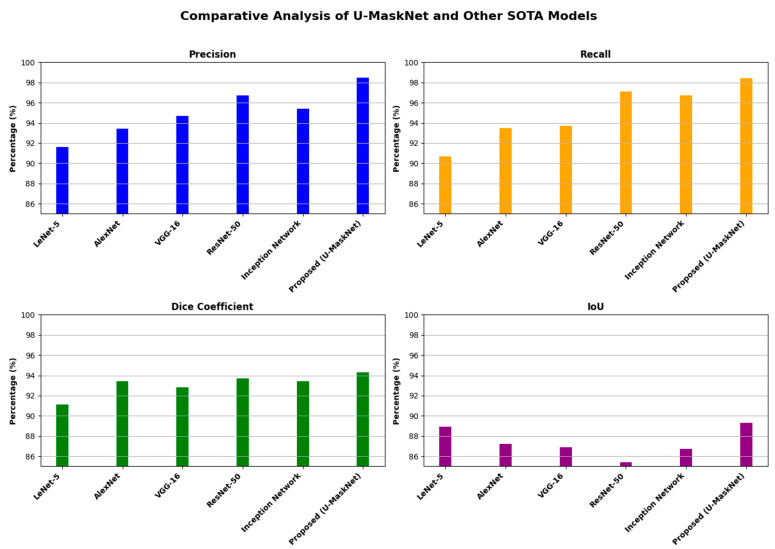
Visualized performance of the proposed U-MaskNet model compared to other state-of-the-art models.

The performance of the proposed U-MaskNet model was compared with some state-of-the-art (SOTA) models [62], as shown in Table 5, to indicate that the proposed method rendered the best results across most parameters. Analyzing the results of the traditional models, such as LeNet-5, AlexNet, VGG-16, ResNet-50, and the Inception Network, we can clearly state that although the precision, recall, Dice, and IoU scores were quite good, they were not as high as the scores for the proposed model, U-MaskNet. Notably, U-MaskNet outperformed ResNet-50 and the Inception Network, which achieved lower Dice and IoU scores compared to our model. The precision and recall of U-MaskNet, 98.5% and 98.4%, respectively, its Dice score of 94.3, and IoU of 89.31, further confirm the practical use of this model and its efficiency in segmenting and detecting gastrointestinal (GI) cancer. Figure 13 represents the visualized performance of the proposed model along with the other SOTA models.

Furthermore, narrow-band imaging (NBI), particularly with magnification endoscopy, has proven to enhance diagnostic accuracy by improving visualization of vascular and mucosal patterns, aiding in the early identification of gastrointestinal lesions [63]. NBI can complement segmentation models by allowing for real-time optical diagnosis, though challenges in standardization and training remain. Integrating NBI with segmentation techniques may provide a robust, accurate diagnostic tool for endoscopic imaging.

The evolution of the polyp segmentation techniques has led to the integration of various methods that improve the performance of the models and the optimization of the boundaries. FMCA-Net avoids over-relying on any feature through a modular design and enhances the edge distinction and achieves superior generalization [64]. Segmentation performance is improved when SAM incorporates models such as DeepLabv3+ through mask fusion, with better performance across datasets [65]. BCL-Former adopts both local enhancement and balanced constraints for the efficient management of polyp diversities and shows superior performance against benchmarks [66]. Last but not least, combinations of convolutional and transformer networks achieve a high level of segmentation performance, possible through a number of configurations, such as loss functions and data augmentation [67]. Altogether, these methods can be regarded as advancements in medical imaging segmentation.

In our future research, we plan to incorporate our current study with other refined methodologies, such as artifact elimination, edge-aware blind deblurring, and saturation correction, to enhance the performance of polyp localization and edge detection. We also intend to research using model quantization and distillation to reduce the model size and parameters, as well as adapt it to compatibility with high-definition medical devices to allow for real-time polyp detection. It can be seen from these changes that a computer-aided diagnosis and treatment system could be further improved to better benefit the patients. The proposed model, which combined U-Net and Mask R-CNN, outperformed the current methods of segmenting GI conditions from the images of endoscopic examinations. Such a model has significant potential to help refine the diagnostic process of GI diseases as well as improve patients’ prognosis and practice effectiveness. The future works will include collecting more data, connecting with the clinical instruments, and using some computer science strategies to fine-tune the model for clinical application.

## 5. Conclusions

In this paper, we introduced a new segmentation model, U-MaskNet, to handle the challenges in detecting and segmenting the various human GI cancers, including dyed lifted polyps, dyed resection margins, esophagitis, normal cecum, normal pylorus, normal Z line, polyps, and ulcerative colitis. Our proposed model, U-MaskNet, integrated U-Net for pixel-wise classification with Mask R-CNN for instance segmentation, effectively addressing the complexity of GI cancer imaging by tackling all relevant aspects. We utilized the Kvasir dataset, which includes a wide variety of GI cancer endoscopic images, comprising 8000 high-quality images. The experimental result showed that our proposed model outperformed well-known models, such as DeepLabv3+, FCN, and DeepMask, as well as state-of-the-art (SOTA) models, including LeNet-5, AlexNet, VGG-16, ResNet-50, and the Inception Network. Regardless of the limited training and evaluation (only 50 epochs), the proposed model consistently achieved high precision, recall, and Dice coefficients with minimal loss across both training and evaluation phases. We performed classification and segmentation tasks on the test dataset and validated the models with standard assessment metrics, including precision, recall, Dice, and IoU. Our proposed model, U-MaskNet, achieved impressive results on the unseen test dataset, with a precision of 98.5%, recall of 98.4%, Dice score of 94.3%, and IoU of 89.31%. We also presented the segmentation results of the various GI cancers, with bounding boxes illustrating the height and width of each segmented mask by the proposed model. The successful application of our novel model for GI cancer disease segmentation and classification demonstrates its potential clinical benefits. The significant findings of our model exhibited accurate and satisfying results, which may assist medical practitioners in the diagnosis of various GI cancer-related diseases more effectively, enhancing the overall diagnostic capabilities.

Overall, this present study evaluated the use of U-MaskNet for medical image segmentation to provide a baseline for future studies in the field. The outcomes provided a theory supporting the model’s performance in complex segmentation tasks and implied that optimization can help the model maintain even higher levels in the future. It is recommended that more data sources could be incorporated in future work and the model architecture could be fine-tuned for enhanced performance of the identified concern to aid the state of affairs of medical image analysis for GI cancer detection.

## Figures and Tables

**Table 1:** Distribution of dataset categories for GI image segmentation.

**S. No.**	**Category**	**Number of Files**
**0.**	Dyed Lifted Polyps	1000
**1.**	Dyed Resection Margins	1000
**2.**	Esophagitis	1000
**3.**	Normal Cecum	1000
**4.**	Normal Pylorus	1000
**5.**	Normal Z Line	1000
**6.**	Polyps	1000
**7.**	Ulcerative Colitis	1000

**Table 2:** Notations and definitions used in the algorithm.

Symbols	Description
D	Dataset consisting of image–label pairs $\left\{\left(X_{i},Y_{i}\right)\right\}$
∝	Learning rate
T	Total number of epochs
B	Batch size
θ	Model parameters
N	Total number of samples
A	Augmentation function
$\theta_{unet,}$	Parameters of the U-Net model
$\theta_{maskrcnn}$	Parameters of the Mask R-CNN model
$X_{batch}$	Batch of input images
$Y_{batch}$	Batch of ground truth tables
$X_{aug}$	Augmented input images
$Y_{aug}$	Augmented ground truth tables
$S_{unet}$	Segmentation output of U-Net
$R_{rois}$	Region of interest (RoI) proposals from Mask R-CNN
$B_{bbox}$	Bounding boxes from Mask R-CNN
$M_{mask}$	Mask predictions from Mask R-CNN
$F_{unet}$	Multi-scale features from U-Net
$H_{features\_maps}$	Integrated feature maps
$L_{s-unet}$	Segmentation loss for U-Net
$L_{s-maskrcnn}$	Segmentation loss for Mask R-CNN
$L_{bbox}$	Bounding box regression loss
$L_{dice}$	Dice coefficient loss
$L_{total}$	Total loss function
$\nabla_{\theta_{unet}}L_{total}$	Gradient of the total loss with respect to U-Net parameters
$\nabla_{\theta_{maskrcnn}}L_{total}$	Gradient of the total loss with respect to Mask R-CNN parameters

**Table 3:** Comparative analysis of segmentation models based on key metrics.

Evaluation Criteria	DeepLabv3+	FCN	DeepMask	U-MaskNet
**Precision**	87.46	98.46	98.45	**98.85**
**Recall**	87.41	98.39	98.03	**98.49**
**Dice coefficient**	87.43	94.12	94.25	**94.35**
**IoU**	77.70	88.90	89.14	**89.31**
**Loss**	13.26	5.18	5.11	**4.88**
**F1 score**	91.96	98.44	98.25	**98.68**
**AUC ROC**	98.86	99.94	99.93	**99.96**

**Table 4:** Performance comparison of our method with well-known DL models. NA—Not Applicable.

Method	Precision	Recall	Dice	IoU
**PolyPooling** [9]	92.9	94.6	93.7	88.5
**CRCNet** [8]	92.9	94.6	93.7	88.5
**U-Net** [55]	82.9	81.5	79.9	83.2
**U-Net++** [56]	89.3	91.0	88.1	81.7
**ASCNet** [57]	92.2	90.0	91.3	90.4
**PraNet** [58]	91.2	91.3	89.8	83.3
**TGANet** [59]	91.3	91.2	88.8	83.4
**Polyp** [60]	NA	NA	93.1	88.0
**MixPolyp** [61]	NA	NA	85.9	78.5
**Proposed (U-MaskNet)**	**98.5**	**98.4**	**94.3**	**89.31**

**Table 5:** Comparative analysis of the proposed U-MaskNet model with the state-of-the-art methods.

Method	Precision	Recall	Dice	IoU
**LeNet-5**	91.6	90.7	91.1	88.9
**AlexNet**	93.4	93.5	93.4	87.2
**VGG-16**	94.7	93.7	92.8	86.9
**ResNet-50**	96.7	97.1	93.7	85.4
**Inception Network**	95.4	96.7	93.4	86.7
**Proposed (U-MaskNet)**	**98.5**	**98.4**	**94.3**	**89.31**

## Data Availability

The dataset utilized in this work is freely available on the official website: https://datasets.simula.no/kvasir/ (accessed on 15 May 2024).
